# Smoking during Pregnancy: Findings from the 2009–2010 Canadian Community Health Survey

**DOI:** 10.1371/journal.pone.0084640

**Published:** 2014-01-08

**Authors:** Yang Cui, Shahin Shooshtari, Evelyn L. Forget, Ian Clara, Kwong F. Cheung

**Affiliations:** 1 Department of Community Health Sciences, Faculty of Medicine, University of Manitoba, Winnipeg, Canada; 2 Faculty of Nursing, University of Manitoba, Winnipeg, Canada; University of Tolima, Colombia

## Abstract

**Objectives:**

Smoking during pregnancy may cause many health problems for pregnant women and their newborns. However, there is a paucity of research that has examined the predictors of smoking during pregnancy in Canada. This study used data from the 2009–2010 Canadian Community Health Survey (CCHS) to estimate the prevalence of smoking during pregnancy and examine the demographic, socioeconomic, health-related and behavioral determinants of this behavior.

**Methods and Findings:**

The data were obtained from the 2009–2010 CCHS master data file. Weighted estimates of the prevalence were calculated. Multivariable logistic regression was used to determine demographic, socioeconomic, health related and behavioral characteristics associated with smoking behavior during pregnancy. Women living in the Northern Territories had a high rate of smoking during pregnancy (59.3%). The prevalence of smoking during pregnancy was also high among women under 25 years old, of low socioeconomic status, who reported not having a regular medical doctor, being fair to poor in self-perceived health, having at least one chronic disease, having at least one mental illness, being heavy smokers, and being regular alcohol drinkers. Results from multivariable logistic regression revealed that the odds of smoking during pregnancy were decreased with increasing age (odds ratio [OR], 0.95; 95% confidence interval [CI], 0.91–0.99), having a regular family doctor [OR, 0.24; 95% CI, 0.11–0.52], having highest level of family income [OR, 0.09; 95% CI, 0.03–0.29]. Mothers who reported poor or fair self-perceived health [OR, 2.13; 95% CI, 0.96–4.71] and those who had at least one mental illness [OR, 1.81; 95% CI, 1.00–3.28] had greater odds of smoking during pregnancy.

**Conclusions:**

There are a number of demographic, socio-economic, health-related and behavioral characteristics that should be considered in developing and implementing effective population health promotional strategies to prevent smoking during pregnancy, promoting health and well-being of pregnant women and their newborns.

## Introduction

Smoking during pregnancy may cause many health problems for pregnant women and their newborns. There are growing public health concerns about the transfer of contamination such as nicotine monoxide and other harmful chemicals from smoking pregnant women to the growing fetus. In particular, harmful substances in tobacco can affect the function of placental vascular and umbilical artery blood flow, and can pass directly into the fetal bloodstream. As shown in accumulated epidemiological evidence, smoking by the mother during pregnancy is strongly associated with low weight birth (<2500 g) [1], adverse physiological effects such as congenital heart defects [2] and sudden infant death syndrome [3]. Smoking during pregnancy imposes a considerable economic burden on the healthcare system. For example, in the United States, annual costs for intensive neonatal care of low-birth weight infants were $272 million, of which $267 million would not be incurred if maternal smoking was prevented [1].

In Canada, the prevalence of smoking among pregnant women was estimated to be 17% in 2000/01 [4]. The prevalence rate has also been shown to vary across the country. For instance, in the same period, the rates of maternal smoking were 12% and 23% in British Columbia and Alberta respectively [5]–[6]. However, data on the prevalence and risk factors of smoking during pregnancy in recent years is limited in Canada. Only three previous studies [4], [7]–[8] assessed smoking during pregnancy in Canada. However, none of these studies examined how health-related characteristics affect smoking behavior during pregnancy. To develop effective programs for the prevention of smoking during pregnancy, we need to know more about the determinants of this behavior. For example, which characteristics, or behaviors, or health conditions of pregnant women increase their likelihood of smoking during pregnancy?

This study used data for a large sample of Canadians to estimate the prevalence of smoking during pregnancy and to determine the demographic, socio-economic, behavioral and health-related characteristics that are associated with smoking during pregnancy.

## Materials and Methods

The data were obtained from the 2009–2010 Canadian Community Health Survey (CCSH). This survey is a cross-sectional survey that collects information related to health status, healthcare utilization and health determinants for the Canadian population. The CCHS covers approximately 98% of the Canadian population aged 12 and older living in ten provinces and three territories. It excludes those persons living on reserves or crown lands, residing in institutions, and full-time member of the Canadian forces.

The 2009–2010 CCHS consisted of 172,761 selected households, of which 139,841 households agreed to participate in the survey, resulting in an overall household-level response rate of 81.0%. Among these responding households, 139,841 individuals (one per household) were selected to participate in the survey, out of which a response was obtained from 124,870 individuals, resulting in an overall person-level response rate of 89.3%. At the Canadian level, this yields a combined response rate of 72.3% for the CCHS 2009–2010 [9]. However, in some cases (2%) [9], the randomly selected respondents were incapable of completing an interview, so proxy responses were allowed. These responses were excluded from the analysis, because proxy respondents may answer differently from self-respondents resulting in potentially biased population estimates.

In the CCHS 2009–2010 questionnaires, in addition to core content that all provinces and territories included, there were optional content modules chosen by individual provinces and territories. The optional content fulfills the need for data at the health region level. This content, while often harmonized across the province, is unique to each region or province and may vary from year to year. One of the optional content modules in 2009–2010 was about smoking during last pregnancy; this module was chosen by two provinces (Ontario and Alberta) and two territories (Yukon and Nunavut) which together represent approximately 50% of the Canadian population. The analysis for this study was based on the responses from these two provinces and two territories. For the purpose of this study, all women aged 15 to 55 who reported being pregnant in the last five years were included.

### Study Measures

#### Smoking during pregnancy

The dependent variable, smoking during pregnancy, was defined based on responses to a single question: “During last pregnancy, did you smoke daily, occasionally or not at all?” Those who responded “daily” and “occasionally” to this question were classified as smokers during the pregnancy. Those who responded “not at all” to this question were classified as non-smokers during the pregnancy.

#### Socioeconomic and demographic factors

Many research studies have found clear socioeconomic gradients with smoking, such as employment, education, income and marital status [10]. Thus, a number of socioeconomic factors were included in this study. Self-reported household income was classified into four categories in this study, based on the number of people in the household and total household income from all sources in the past 12 months [11]: lowest income, lower-middle income, middle-upper income and highest income. Other socioeconomic and demographic variables included were: age of respondent (in years), education level (<university/university graduate), employment status (had a job last week/did not have a job last week), marital status (married or living as a couple/not living as a couple, who are divorced, separated, or widowed).

#### Health-Related factors

A number of studies have determined that smoking has causal relationship with mental disorders [12], [13], chronic diseases such as diabetes, asthma, heart disease [14], as well as healthcare utilizations [15]. Therefore, based on these literatures and the availability of data from the source survey, the following health-related determinants of smoking during pregnancy were included: self-perceived health (good/poor); self-perceived mental health (good/poor), feeling stressed (not at all to not very stressed versus a bit to extremely stressed), presence of a chronic disease (i.e. had at least one of asthma, heart disease, diabetes, hypertension, arthritis, migraine headache versus no chronic condition); has chronic mental condition (presence of either a mood or anxiety disorder versus no disorder); has regular medical doctor (yes/no).

Two substance-related variables were included in the analysis. A smoking behavior variable was created by classifying respondents into two groups based on the frequency of smoking in 2009–2010. Those who usually smoked 20 or more cigarettes a day were defined as heavy smokers. Those who smoked less than 20 cigarettes were classified as light smokers. A drinking behavior variable: regular alcohol drinker (yes/no) was created by grouping individuals into two groups based on the frequency of their drinking behavior.

### Analytical Techniques

To address the research objectives, the following analyses were conducted: 1) The prevalence of smoking during the pregnancy was estimated using weighted frequencies; 2) A series of bivariate analyses examined the cross-sectional association between each characteristic listed above and smoking behavior during the pregnancy. Results of these bivariate analyses informed the multivariate analyses which involved the development of a multivariable logistic regression model. 3) Multivariable logistic regression examined the extent to which individuals’ socioeconomic, demographic, health-related, and behavioral factors were associated with smoking behavior during pregnancy of the target population. Significant predictors were identified based on the adjusted odds ratios and their 95% confidence intervals (CIs). Data analyses were performed using SAS v9.2 statistical software and SUDAAN version 10.0.1. For all analyses, the bootstrapping procedure was performed in SUDAAN to obtain design-based estimates for all model parameters. These analyses used the bootstrap weights that were provided by Statistics Canada and contained in the CCHS master data file.

### Ethics Statement

The University of Manitoba Research Ethics Board expedites approval for secondary analysis of Statistics Canada data conducted in the Manitoba Research Data Centre. It has waived the need for written informed consent. This study was submitted to Statistics Canada for approval to access National Community Health Survey master data files at the Manitoba Research Data Centre. A number of measures employed to protect the confidentiality of the data; for example, disclosure avoidance analysis was conducted by the data analyst from Statistics Canada on all data released.

## Results

The study population consisted of 369,547 women aged 15 to 55 who gave birth in the last five years and answered yes or no to the question “During your last pregnancy, did you smoke daily, occasionally or not at all?”. The estimated prevalence of smoking during pregnancy was 23% overall (Table 1). The estimated prevalence of smoking during pregnancy was higher in the Northern Territories (59.3%) compared to the rates in Alberta (34.8%) and Ontario (18.5%).

**Table 1:** Weighed prevalence (%) of smoking during pregnancy by Provinces and Territories.

	Weighted Prevalence	Lower 95%CI	Upper 95% CI
Ontario	18.5	15.4	22.2
Alberta	34.8	28.2	42.0
NorthernTerritories	59.3	48.6	69.2
Overall	23.0	19.2	26.5

Data source: 2009–2010 Canadian Community Health Survey.

Table 2 displays the weighted prevalence of smoking during pregnancy in terms of the selected socioeconomic, demographic, health-related and behavioral characteristics. The prevalence of smoking during pregnancy was higher for women who were under 25 years old (38.6%), having lowest household income (59.6%), less than university educated (18.8%), not living as a couple (42.1%), and who did not have a job recently (24.9%). These findings suggest that individuals in lower socioeconomic groups are at an increased risk for unhealthy behaviors such as smoking during pregnancy. In addition, smoking during pregnancy was more prevalent among women who did not have a regular medical doctor (50.5%), rated their overall health as being fair to poor (43.1%), rated their mental health as being fair, or poor (37.7%), had at least one chronic disease (30.8%), had at least one mental disease (35.3%), felt stressed (23.2%), were heavy smokers (86%) and regular alcohol drinkers (27.8%).

**Table 2:** Weighed prevalence (%) of smoking during pregnancy according to socioeconomic, demographic, behavioral, health-related factors.

	Factor	WeightedPrevalence (%)	Lower95%CI	Upper95% CI	*P*-value*
**Demographic**					
Age group	15–24	38.6	30.3	47.5	<.001
	25–34	24.7	20.6	29.2	
	35 and older	15.1	15.4	22.0	
Race	Caucasian	21.1	18.0	24.5	0.04
	Visible minority	31.4	22.8	41.4	
**Socioeconomic**					
Total household income	Lowest income	59.6	34.6	80.4	<.001
	Lower-middle income	45.9	31.4	61.2	
	Middle income	36.2	26.1	47.7	
	Upper-middle	22.7	17.0	29.7	
	Highest income	9.1	6.6	12.6	
	Missing	29.5			
Education	<University	18.8	15.6	22.5	<.001
	> = University	5.1	1.8	13.5	
	Missing	29.8			
Employment	Did not have a job last week	24.9	20.9	29.3	0.30
	Had a job last week	21.4	16.8	26.9	
Marital status	Married or living as a couple	17.0	14.4	19.9	<.001
	Not living as a couple	42.1	33.9	50.8	
**Health-related**					
Has a regular medical doctor	Yes	20.6	17.5	24.1	<.001
	No	50.5	37.4	63.6	
Self-perceived health	Good to excellent	21.7	18.4	25.3	0.002
	Fair to poor	43.1	31.5	55.5	
Self-perceived mental health	Good to excellent	22.2	19.1	26.5	0.03
	Fair to poor	37.7	25.8	51.2	
Has chronic disease†	At least one	30.8	24.6	37.9	0.004
	No	19.4	16.3	22.9	
Stress	Not at all to not very stressful	22.5	16.5	29.8	0.85
	A bit to extremely stressful	23.2	19.7	27.1	
Has chronic mental disease‡	At least one	35.3	27.8	43.5	<.001
	No	21.0	17.6	24.7	
**Behavioral**					
Frequency of smoking	Light smoker	61.3	54.2	68.0	<.001
	Heavy smoker	86.0	74.1	92.8	
Regular alcohol drinker	Yes	27.8	22.6	33.6	0.02
	No	19.6	15.9	23.8	

Data source: 2009–2010 Canadian Community Health Survey *Chi-square test;

One of Heart disease, Asthma, Diabetes, Arthritis, Hypertension, Migraine headache;

One of Depression, Anxiety.

Given the binary nature of the dependent variable (smoking during pregnancy: yes/no) multivariable logistic regression modeling was performed to determine the significant predictors of the outcome variable. All of the factors that showed a significant bivariate association with the outcome variable were included in the regression model. Controlling for the effects of all the other factors, five factors remained statistically significant in the multivariable logistic regression model: age, total household income, self-perceived health, having a regular medical doctor, and having a chronic mental illness. The adjusted odds ratios and their 95% CIs are presented in Table 3.

**Table 3:** Weighed multivariable logistic regression.

	Factor	OR (95% CI)	*p*-value
Age		0.95 (0.91, 0.99)	0.01
Has a regularmedical doctor	Yes	0.24 (0.11, 0.52)	<.001
	No	1.00	
Self-perceivedhealth	Good to excellent	1.00	0.06
	Fair to poor	2.13 (0.96, 4.71)	
Has chronicmental disease	Yes	1.81 (1.00, 3.28)	0.05
	No	1.00	
Total householdincome	Lowest income	1.00	<.001
	Lower-middleincome	0.44 (0.12, 1.62)	
	Middle income	0.34 (0.10, 1.13)	
	Upper-middle	0.20 (0.06, 0.69)	
	Highest income	0.09 (0.03, 0.29)	

Data source: 2009–2010 Canadian Community Health Survey.

Total household income, which is considered to reflect socioeconomic status, was negatively associated with smoking during pregnancy. Women were less likely to smoke during pregnancy with increasing age [OR = 0.95, 95% CI: 0.91, 0.99], or if they have a regular family physician [OR = 0.24, 95% CI: 0.11, 0.52]. The results also revealed that poor self-perceived health was associated with an increased risk of smoking during pregnancy. In fact, women who reported poor to fair self-perceived health were more than twice as likely to smoke during pregnancy [OR = 2.13, 95% CI: 0.96, 4.71] compared to those with good to excellent self-perceived health. Furthermore, health-related conditions such as suffering a chronic mental condition including depression and/or anxiety were found to be associated with smoking during pregnancy [OR = 1.81, 95% CI: 1.00, 3.28].

## Discussion

This study examined the prevalence and characteristics of smoking during pregnancy among women in two provinces and two territories. The results showed the prevalence rate of maternal smoking was relatively high in Northern Territories (59.3%). This high prevalence may be partly due to the fact that tobacco has traditionally been used in many ceremonies, rituals and medicine in First Nations [16]. This finding may also indicate that there is insufficient investment in public health campaigns such as tobacco harm reduction in the remote territories compared to the other provinces. Therefore, cost-effective interventions should be tailored to meet the needs of pregnant women in the Northern Territories.

Since previous research only investigated socioeconomic and demographic factors related to smoking during pregnancy, this study attempted to fill a gap in the literature by examining how health-related and behavioral factors are associated with the behavior of smoking during pregnancy. This study found that having a regular medical provider was associated with a decreased risk of smoking during pregnancy. This result illustrates that healthcare providers, such as family physicians, may play a vital role in helping their patients to stop smoking. Therefore, the increased use of family physicians or general practitioner services in remote areas might be effective in raising awareness of harmful tobacco use and reducing the risk of smoking during pregnancy. As an example, smoking cessation programs in clinics can provide education on the effects of smoking during pregnancy, and smoking cessation counseling by healthcare professionals can bolster the effects of such programs and raise awareness about the risks associated with smoking during pregnancy. In addition, this study found that mental health and poor self-perceived health are significant factors that affect the smoking behavior during pregnancy. Therefore, any intervention should ensure that pregnant women with mental illness can access the anti-smoking services.

The findings from this study are comparable with the previous Canadian studies. Compared to other studies, the overall prevalence rate of smoking during pregnancy was relatively stable. For example, Connor and MccIntyre (1999) [8] estimated the prevalence rate of smoking during pregnancy in 1994 was 23.7%, while Millar and Hill (2004) [4] estimated the prevalence to be 17% using data from the 2000/01 CCHS. These authors also found that rates of smoking during pregnancy varied by province, with lowest rates in Ontario (14%) and British Columbia (14%). Al-Sahab et al. (2010) [7] used the Maternal Experience Survey (MES) to estimate the prevalence rate of smoking during pregnancy (10.5%) among Canadian women who had singleton live births in 2006. They also found that the Northern Territories had the highest prevalence rate (39.4%) among pregnant women. The findings also are consistent with other studies that reported age (<25), low income status, having mental illness, and self-perceived health to be associated with higher smoking prevalence in Canada [4], [7], [12]–[13], [17]–[20]. However, the limitations of comparing the results from different surveys should be acknowledged. For example, MES had response rate of 75.2% and targeted Canadian women who had singleton live births within three months in 2006. The 2009–2010 CCHS had 89.3% response rate, but only four provinces and territories performed the optional content on smoking during pregnancy.

The findings of this study have several limitations that can affect their interpretation. First, the CCHS data are self-reported; thus recall bias is unknown. A related limitation is that, for many of the items in the survey, individuals are asked to remember if they have ever had a chronic condition or mood/anxiety disorder, and their recall could be incorrect. Second, the CCHS survey covers 98% of the total population, but information from the other 2% population was excluded. This missed population may include women residing in shelters or the homeless who are at a high risk of drug abuse problems and a high risk of smoking during pregnancy [21]. This may result in an underestimate of the true prevalence of smoking during pregnancy in the population. Third, the survey questions regarding smoking during pregnancy do not differentiate levels of exposure to smoking during the trimesters of pregnancy. This prevents a further analysis on patterns of smoking during pregnancy. Finally, the CCHS survey module on smoking during pregnancy was an optional module, and only four provinces and territories opted in to this module. As a result, the estimated prevalence rate cannot represent the whole country.

## Conclusions

In short, findings from this study might enhance the current knowledge of personal characteristics that are associated with increased likelihood of smoking during pregnancy. This is important information which can be used by those involved in public health education and promotion to develop effective strategies for prevention of smoking during pregnancy or promoting smoking-cession among pregnant women. On the basis of these findings, this study can guide healthcare providers and policy makers to promote health within the target population. By doing so, more effective and efficient interventions can be tailored to their needs.

## References

[1] Oster G, Delea TE, Colditz GA (1988) Maternal smoking during pregnancy and expenditures of neonatal health care. Am J Prev Med 4 (4): 216–9. 3139017

[2] Alverson CJ, Strickland MJ, Gilboa SM, Correa A (2011) Maternal smoking and congenital heart defects in the Baltimore-Washington infant study. Pediatrics 127 (3): e647–53. 10.1542/peds.2010-1399 21357347

[3] Haustein KO (1999) Cigarette smoking, nicotine and pregnancy. Int J Clin Pharmacol Ther 37(9): 417–27. 10507240

[4] Millar WJ, Hill G (2004) Pregnancy and smoking. Health Rep 15 (4): 53–6. 15346729

[5] Alberta Health (2011) Alberta reproductive health pregnancies & Births, table update 2011. Available: http://www.health.alberta.ca/documents/reproductive-health-2011.pdf. Accessed 17 July 2012.

[6] British Columbia Perinatal Database Registry (2006) Annual Report 2006. Available: http://www.bcphp.ca/sites/bcrcp/files/Annual%20Report%202006.pdf. Accessed 9 July 2012.

[7] Al-Sahab B, Saqib M, Hauser G, Tamim H (2010) Prevalence of smoking during pregnancy and associated risk factors among Canadian women: A national survey. BMC Pregnancy Childbirth 10: 24 10.1186/1471-2393-10-24 20497553 PMC2885995

[8] Connor SK, McIntyre L (1999) The socio-demographic predictors of smoking cessation among pregnant women in Canada. Can J Public Health 90(5): 352–5. 10570583 10.1007/BF03404527 PMC6979916

[9] Statistics Canada (2011) Canadian community health survey (CCHS) -annual component. User guide 2010 and 2009–2010 microdata files. Ottawa: Statistics Canada.

[10] The Canadian Institute for Health Information (2003) Women’s health surveillance report. Ottawa: The Canadian Institute for Health Information.

[11] Statistics Canada (2010) Canadian Health Measures Survey cycle 1 data dictionary. Ottawa: Statistics Canada.

[12] Lawrence D, Mitrou F, Zubrick SR (2009) Smoking and mental illness: results from population surveys in Australia and the United States. BMC Public Health 9: 285 10.1186/1471-2458-9-285 19664203 PMC2734850

[13] Leonard S, Adler KE, Benhammou K, Berger R, Breese CR, et al (2001) Smoking and mental illness. Pharmacol Biochem Behav 70(4): 561–70. 11796154 10.1016/s0091-3057(01)00677-3

[14] Public Health Agency of Canada (2007) Tobacco use. Available: http://www.phacaspc.gc.ca/publicat/2007/lbrdc-vsmrc/tobacco-tabagisme-eng.php. Accessed 9 July 2012.

[15] Kahende JW, Adhikari B, Maurice E, Rock V, Malarcher A (2009) Disparities in healthcare utilizations by smoking status-NHANES 1999–2004. Int J Environ Res Public Health 6(3): 1095–1106 10.3390/ijerph6031095 19440435 PMC2672402

[16] Health Canada (2010) First Nations Inuit and Aboriginal Health. Available: http://www.hcsc. gc.ca/fniah-spnia/substan/tobac-tabac/index-eng.php#use. Accessed 9 July 2012.

[17] Nagahawatte NT, Goldenberg RL (2008) Poverty, maternal health, and adverse pregnancy outcome. Ann N Y Acad Sci. 1136: 80–85. 10.1196/annals.1425.016 17954684

[18] Schneider S, Schütz J (2008) Who smokes during pregnancy? A systematic literature review of population-based surveys conducted in developed countries between 1997–2006. Eur J Contracept Reprod Health Care. 13(2): 138–47 10.1080/13625180802027993 18465475

[19] Heaman MI, Chalmers K (2005) Prevalence and correlates of smoking during pregnancy: A comparison of aboriginal and non-aboriginal women in Manitoba. Birth 32(4): 299–305. 16336371 10.1111/j.0730-7659.2005.00387.x

[20] Muhajarine N, D’Arcy C, Edouard L (1997) Prevalence and predictors of health risk behaviours during early pregnancy: Saskatoon pregnancy and health study. Can J Public Health 88(6): 375–9. 9458562 10.1007/BF03403909 PMC6990349

[21] Fischer PJ, Breakey WR (1991) The epidemiology of alcohol, drug, and mental disorders among homeless persons. Am Psychol 46(11): 1115–28. 1772149 10.1037//0003-066x.46.11.1115

